# Supplementary material for: Causal Relationship between Aging and Anorexia Nervosa: A White-Matter-Microstructure-Mediated Mendelian Randomization Analysis
Source: Biomedicines. 2024 Aug 16;12(8):1874. doi: 10.3390/biomedicines12081874 (PMC11351342; doi:10.3390/biomedicines12081874)
Supplement: Supplementary file 1 [file biomedicines-12-01874-s001.zip › Supplementary Figure/Figure S9/Leaveoneout plot of MR analysis for ubm-b-1847 to AN.pdf]

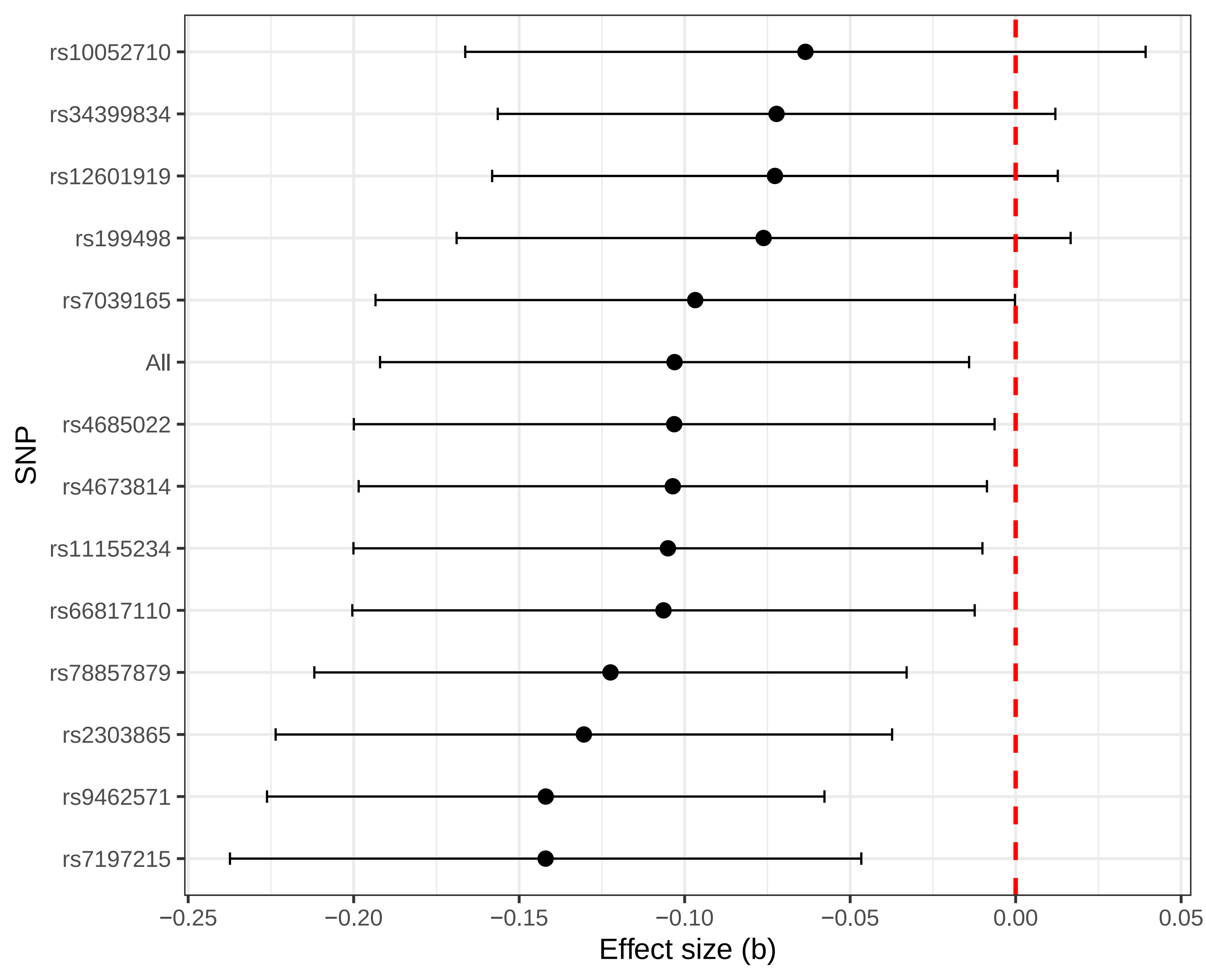
rs10052710
rs34399834
rs12601919
rs199498
rs7039165
All
rs4685022
rs4673814
rs11155234
rs66817110
rs78857879
rs2303865
rs9462571
rs7197215
SNP
−0.25
−0.20
−0.15
−0.10
−0.05
0.00
0.05
Effect size (b)
